# Optic Nerve Glioma, Plexiform Neurofibroma, and Secondary Glaucoma in a Child With a Rare NF1 Variant (p.Gln83Ter): A Case Report

**DOI:** 10.7759/cureus.95957

**Published:** 2025-11-02

**Authors:** Kainat Chaudhary, Suneeta Dubey

**Affiliations:** 1 Department of Glaucoma, Dr. Shroff's Charity Eye Hospital, New Delhi, IND

**Keywords:** neurofibromatosis type 1 (nf1), nf1 gene variant (p.gln83ter), optic nerve glioma, plexiform neurofibroma, secondary glaucoma

## Abstract

Neurofibromatosis type 1 (NF1) is a common autosomal dominant disorder with highly variable expression, ranging from cutaneous findings, such as café-au-lait macules, to severe ophthalmic and neurological complications. Among its ocular manifestations, optic pathway gliomas and plexiform neurofibromas are well recognized, while secondary glaucoma, though less frequent, can significantly impair vision and quality of life. We present the case of a four-year-old girl with NF1 who developed a unique clustering of optic nerve glioma, plexiform neurofibroma, and secondary glaucoma, underscoring the spectrum and severity of ocular involvement in this condition. Genetic analysis identified a rare nonsense NF1 variant (p.Gln83Ter), also found in other affected family members, providing valuable insight into genotype-phenotype correlation. This case highlights several important points such as the need for a high index of suspicion in young children presenting with unusual or coexisting ocular manifestations, the utility of early genetic confirmation for diagnosis and family counseling, and the importance of coordinated, multidisciplinary care to address the diverse and progressive complications of NF1.

## Introduction

Neurofibromatosis type 1 (NF1) is an autosomal dominant neurocutaneous disorder [1] with an incidence of approximately 1:3,500 births [2]. More than 3,000 *NF1* variants have been described, but most are family-specific [1]. A study from India found that nonsense variants (~36%) were the most frequent mutation type in *NF1*, followed by frameshift, splice site, and deletion mutations. Three variants - c.5269-1G>C, c.1541_1542delAG, and c.6853_6854insA - were reported to occur in more than one patient; however, even these were not frequent and remained relatively uncommon within the cohort [3].

Café-au-lait macules and cutaneous neurofibromas are among the most recognizable hallmarks of NF1. Ocular complications such as optic pathway gliomas, plexiform neurofibromas, and secondary glaucoma can pose unique diagnostic and therapeutic challenges especially when they occur together in a young child. Ocular manifestations such as optic pathway gliomas and secondary glaucoma may threaten vision [4].

Plexiform neurofibromas, often considered a hallmark of NF1, are slow-growing but infiltrative, with the potential to distort normal orbital and periocular structures [4]. The underlying mechanism of NF1-associated glaucoma is multifactorial, with potential causes including direct infiltration of the anterior chamber angle by neurofibromas, secondary angle closure resulting from neurofibromatous thickening of the ciliary body and choroid, fibrovascularization leading to synechial angle closure and neovascular glaucoma, and developmental angle abnormalities [5].

In this report, we describe the case of a four-year-old girl with NF1 who developed optic nerve glioma, periocular plexiform neurofibroma, and secondary glaucoma. Genetic analysis revealed a rare nonsense *NF1* variant (p.Gln83Ter), resulting in premature truncation of the neurofibromin protein.

This case is remarkable as it brings together an unusual constellation of vision-threatening complications in a very young child, all associated with a rare *NF1* variant. Such complex presentations highlight the need for timely recognition, coordinated multidisciplinary care, and thoughtful consideration of possible genotype-phenotype associations in NF1. By reporting this case, we aim to expand clinical awareness, add to the limited literature on rare *NF1* mutations, and draw attention to their potential role in shaping atypical or severe disease patterns.

## Case presentation

A four-year-old girl presented with swelling over the left eyebrow and proptosis of the left eye. At one year of age, a brown patch over the left brow had gradually progressed into a nodule, for which excision biopsy was performed. The histopathology report was not available, as it had not been provided by the patient’s attendant. She later developed elevated intraocular pressure (IOP) in the left eye and underwent goniotomy in 2023.

**Figure 1:**
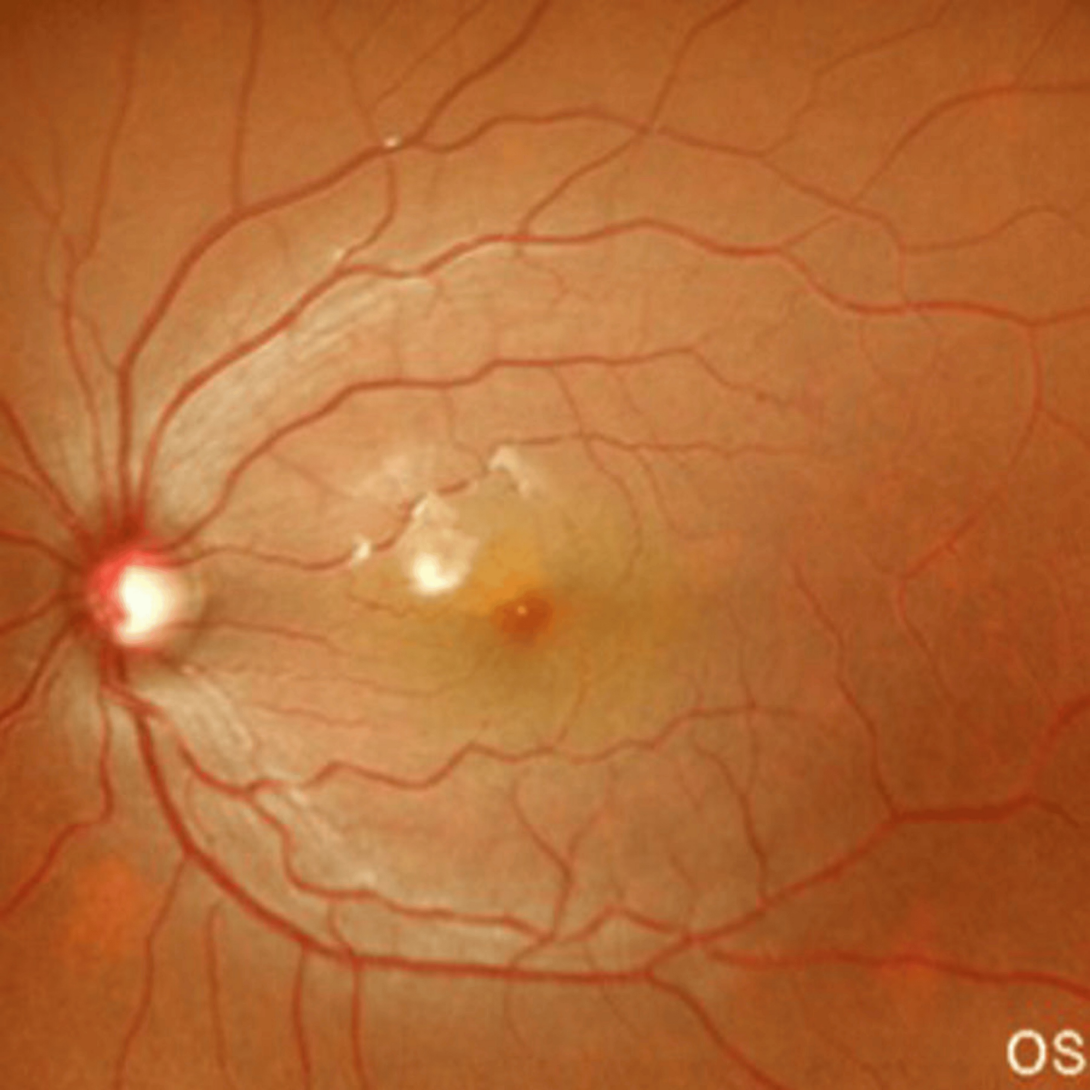
Fundus photograph showing left eye cup (CDR of 0.6:1, IR sloping rims, good RNFL) CDR, cup-to-disc ratio; IR, inferior rim; RNFL, retinal nerve fiber layer

On examination, her visual acuity was 6/6 in the right eye and 6/18 in the left eye (−3.50 DSph/−2.75 DCyl). IOP measured 17 mmHg in the right eye and 28 mmHg in the left eye on three antiglaucoma medications. Fundus evaluation of the left eye revealed a cup-to-disc ratio (CDR) of 0.6:1 with inferior rim thinning (Figure 1). The white-to-white (WTW) corneal diameter measured 11 mm in the right eye and 12.8 mm in the left eye, with the latter finding being suggestive of buphthalmos.

**Figure 2:**
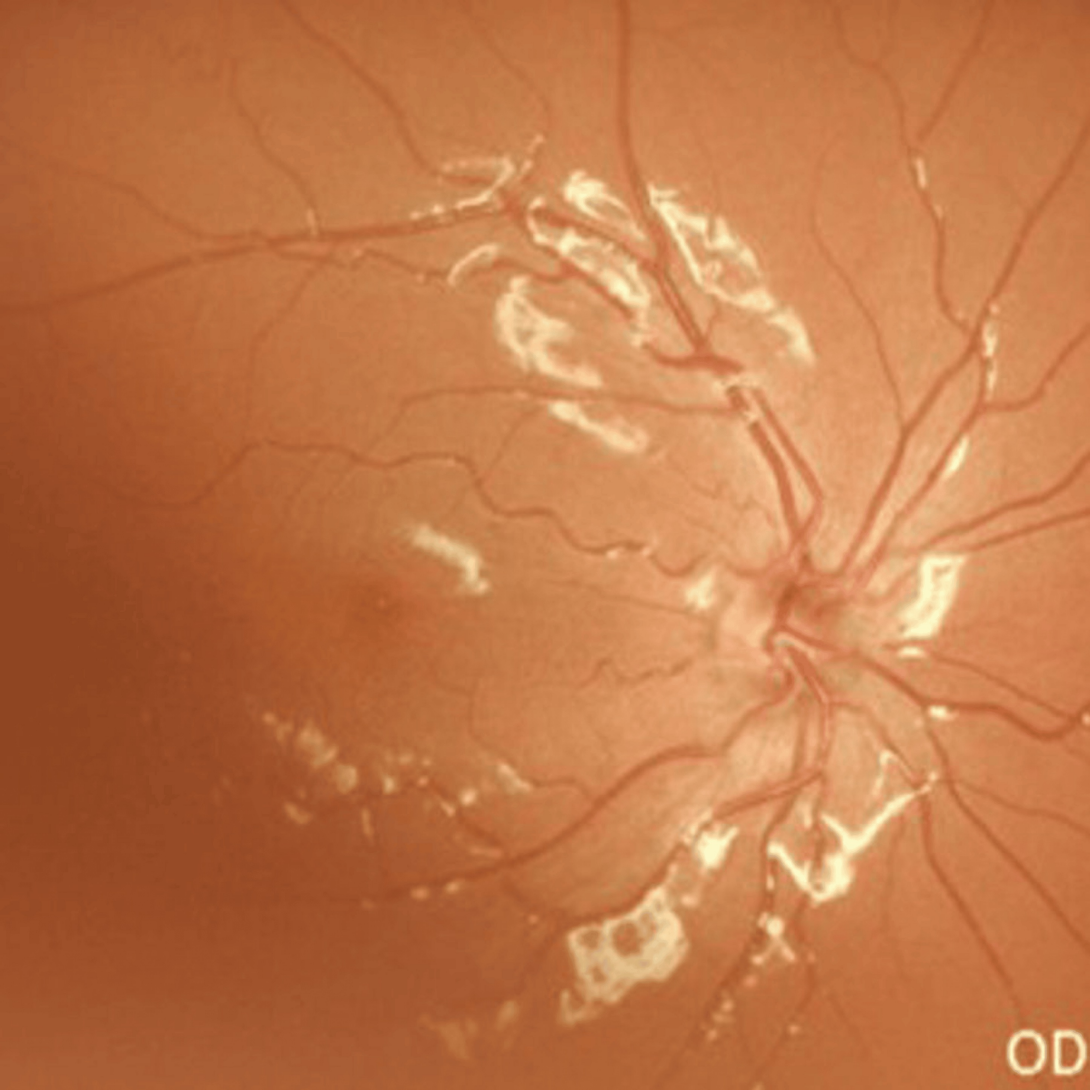
Fundus photograph showing right eye (CDR of 0.2:1, good RNFL visibility) CDR, cup-to-disc ratio; RNFL, retinal nerve fiber layer

The right eye showed a CDR of 0.2:1 with well-defined retinal nerve fiber layer striations (Figure 2). Gonioscopy revealed open angles in the right eye and intermittent peripheral anterior synechiae (PAS) involving all quadrants in the left eye.

**Figure 3:**
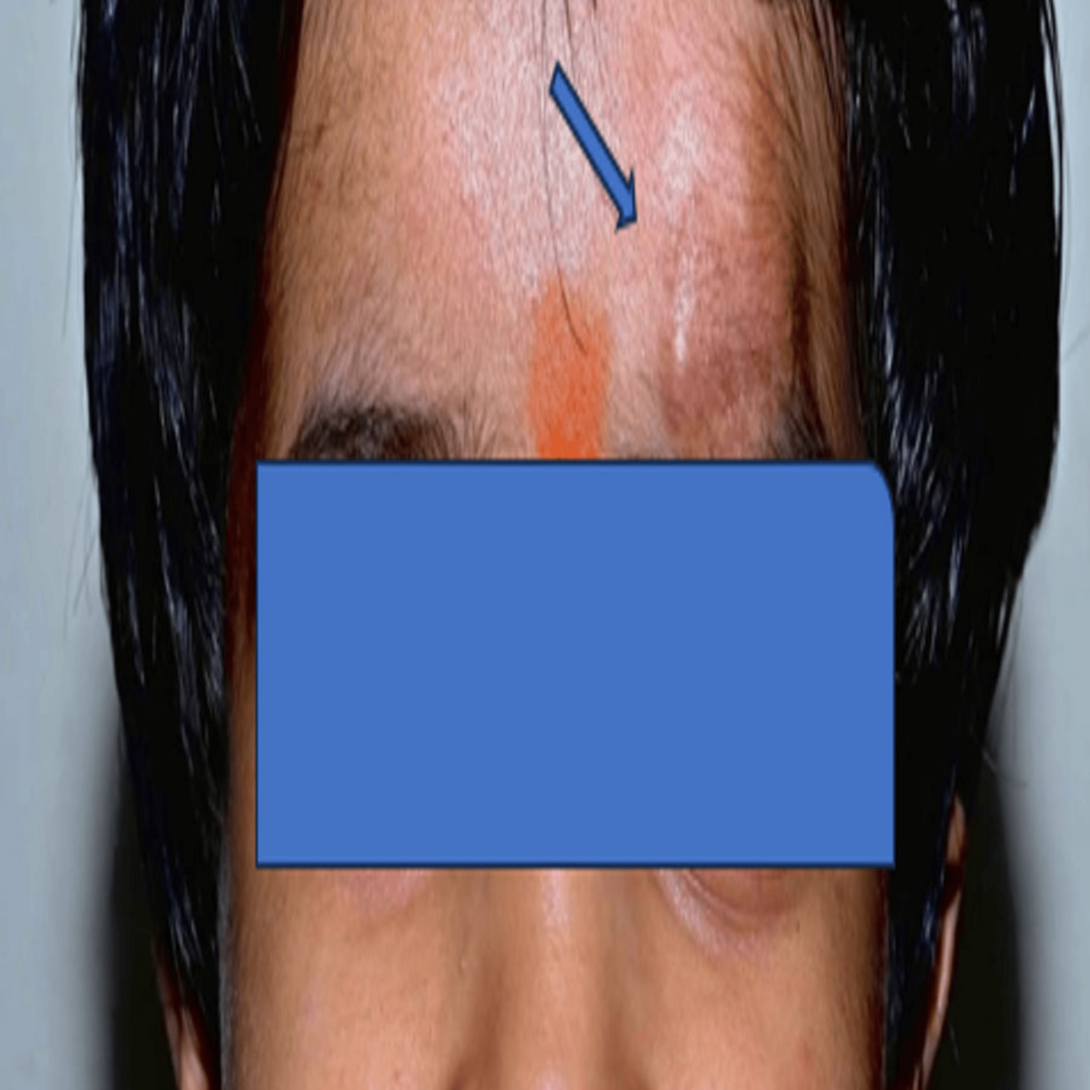
Plexiform neurofibroma over the left supraorbital ridge

On clinical examination, a soft, irregular, plexiform neurofibroma was observed over the left supraorbital ridge (Figure 3).

**Figure 4:**
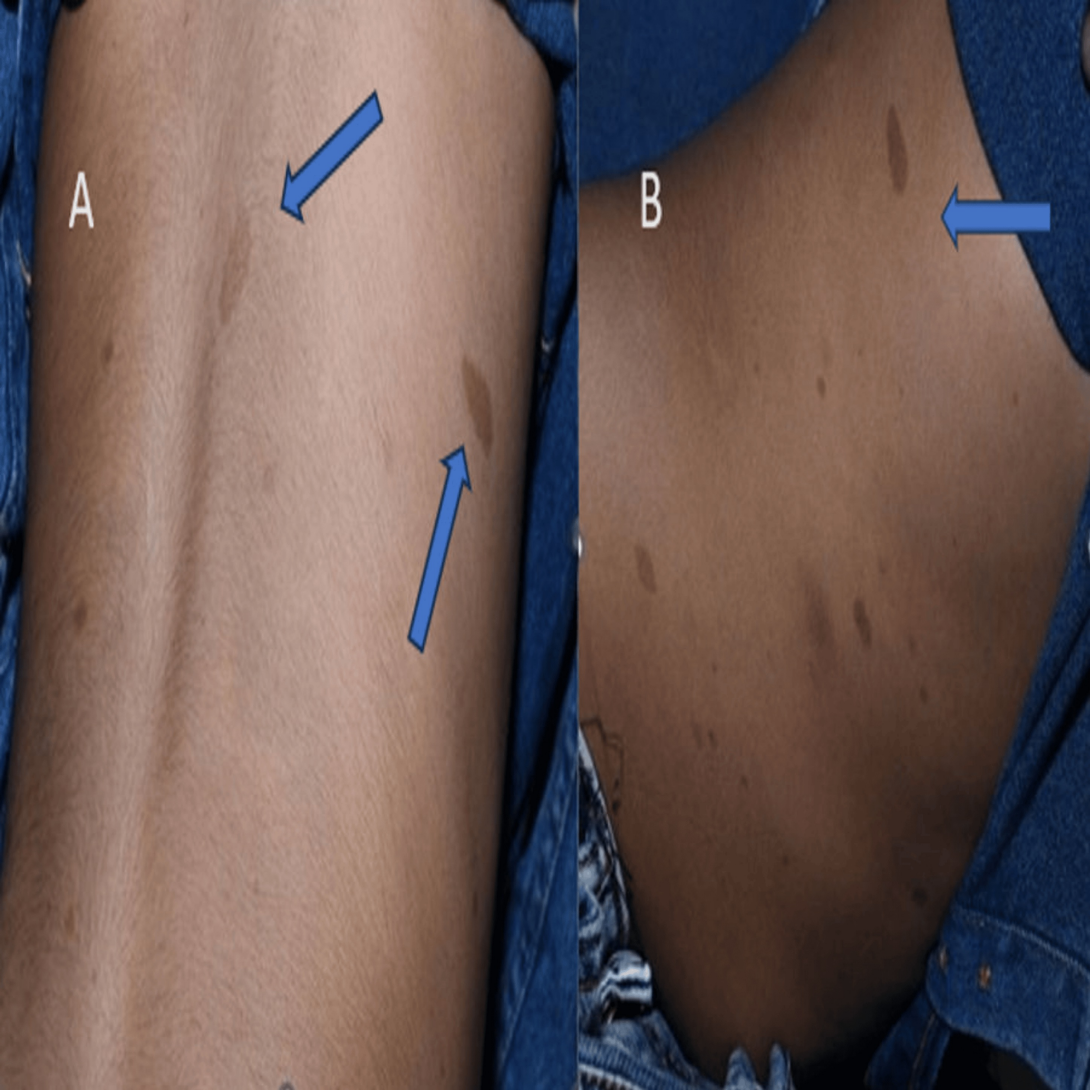
Cafe-au-lait macules on the back and right lumbar region (A) Cafe-au-lait macules noted on the back. (B) Cafe-au-lait macules on the right lumbar region (right flank/lateral aspect of the abdomen).

Café-au-lait macules were observed on the back, abdomen, and lateral aspects of the abdomen (Figure 4).

**Figure 5:**
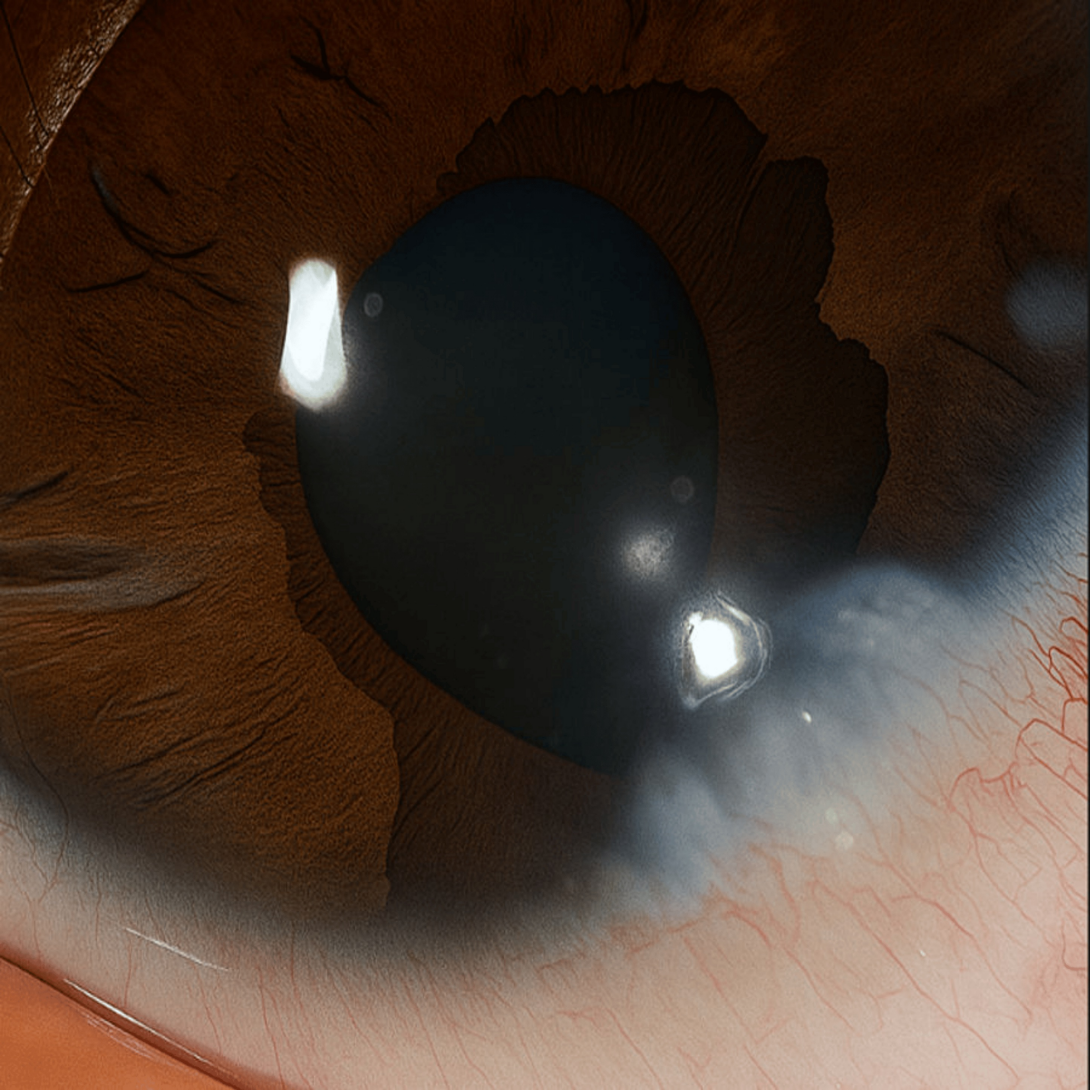
Left eye showing irregular and eccentric pupil with ectropion uveae

On slit-lamp examination, the left eye demonstrated ectropion uveae associated with an irregular and eccentrically positioned pupil (Figure 5).

**Figure 6:**
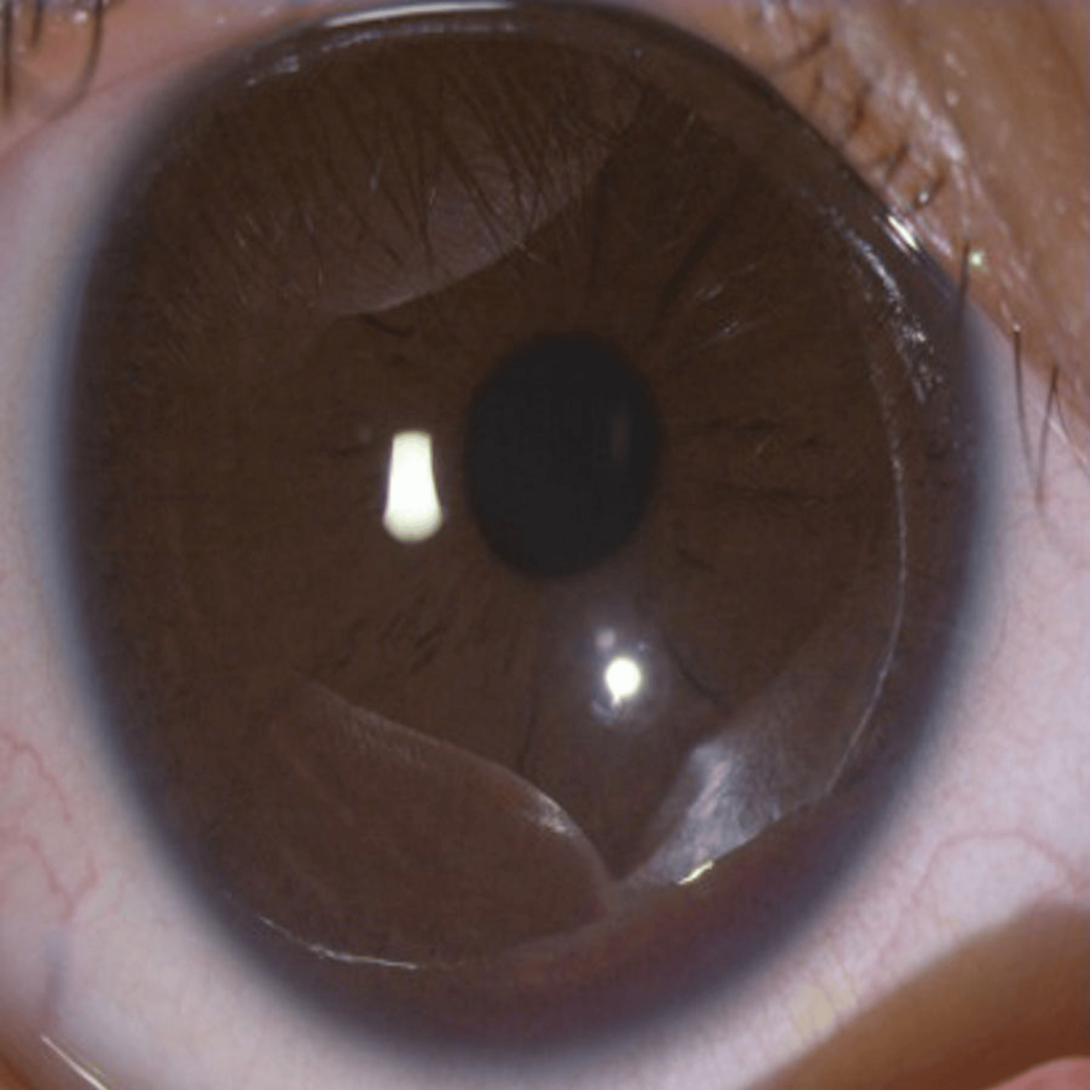
Right eye (normal)

In contrast, the right eye exhibited a clear cornea, a round and regular pupil, and a well-formed anterior chamber (Figure 6).

**Figure 7:**
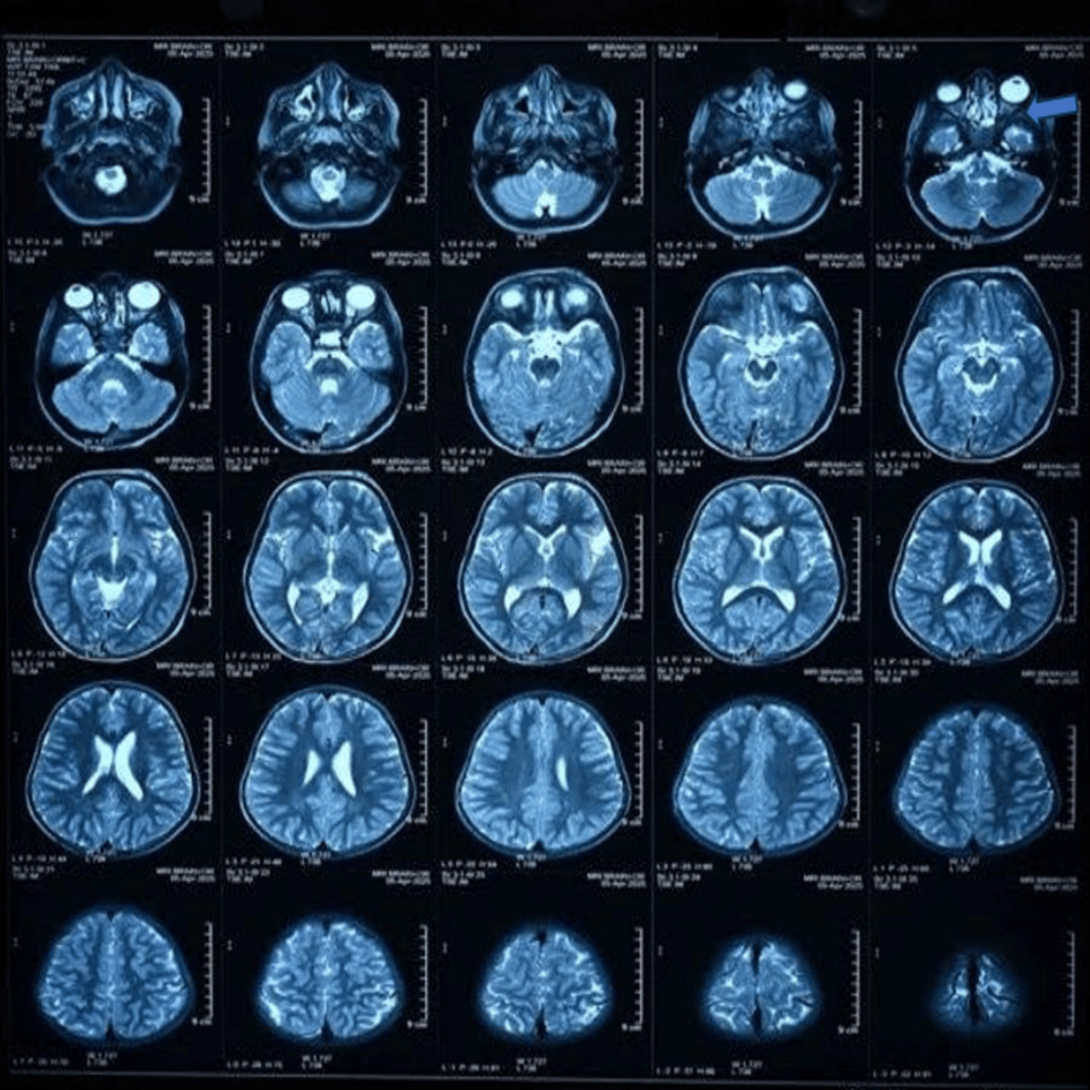
Axial T2-weighted MRI scan of the brain and orbits.

Axial T2-weighted MRI of the brain and orbit was performed, which revealed a mildly enlarged left globe with increased anteroposterior dimension (buphthalmos). There was diffuse enlargement of the intraorbital and prechiasmatic segments of both optic nerves, with fullness of the optic nerve sheaths indenting the posterior globe at the optic nerve head, suggestive of optic gliomas. A cutaneous-subcutaneous plexiform neurofibroma was noted in the left supraorbital ridge (Figure 7).

**Figure 8:**
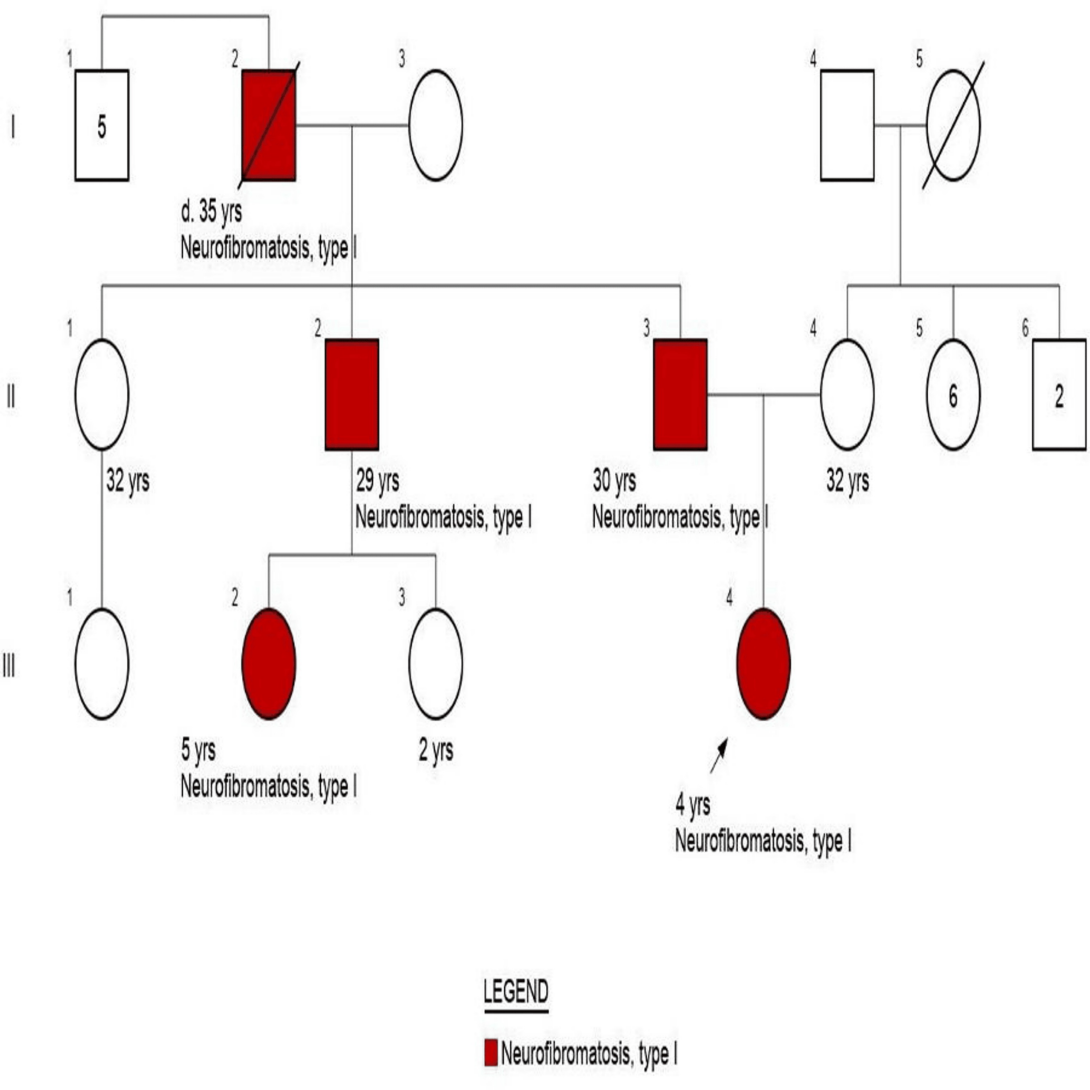
Pedigree charting of the family

Detailed history elicited neurofibromatosis in the patient’s grandfather, paternal uncle, paternal cousin, and father. She was referred to the genetics clinic for evaluation, and a pedigree chart was constructed (Figure 8).

A heterozygous *NF1* nonsense variant (c.247C>T; p.Gln83Ter) in exon 3 (chr17:31159052C>T) was identified by next-generation sequencing and confirmed by Sanger sequencing. This mutation introduces a premature stop codon, resulting in a truncated, nonfunctional neurofibromin protein. Segregation analysis showed the variant in affected family members, consistent with autosomal dominant inheritance (Table 1).

**Table 1:** Genetic test report (NGS of the proband) NGS, next-generation sequencing

Parameter	Result
Gene	*NF1 *(neurofibromin 1)
Chromosomal location	17q11.2
Reference transcript	NM_000267.3
Nucleotide change	c.247C>T
Protein change	p.Gln83Ter (Q83*)
Variant type	Nonsense mutation
Zygosity	Heterozygous
Inheritance pattern	Autosomal dominant
Pathogenicity	Pathogenic (ACMG/ClinVar)
Predicted effect	Truncated protein → loss of function
Database references	ClinVar: RCVxxxxx dbSNP: rsxxxxxx
Phenotype correlation	Variant is consistent with the patient’s clinical presentation (optic nerve glioma, plexiform neurofibroma, secondary glaucoma)

## Discussion

NF1 is caused by pathogenic variants in the *NF1 *gene on chromosome 17q11.2, which encodes neurofibromin, which is a large tumor-suppressor protein that negatively regulates RAS signaling and restrains cell proliferation [1]. Loss of neurofibromin function through germline *NF1 *mutations predisposes to a spectrum of tumors and hamartomas, most notably plexiform neurofibromas and optic pathway gliomas (OPGs), with the latter occurring in roughly 15-20% of children with NF1 and causing visual loss in a subset [6]. Mechanistically, haploinsufficiency of NF1 and subsequent somatic loss of the second NF1 allele in affected cells leads to hyperactive Rat sarcoma - Mitogen activate protein kinase (RAS-MAPK) signaling, promoting abnormal Schwann-cell and glial proliferation that underlies plexiform neurofibroma and OPG formation [7].

Glaucoma associated with NF1 is uncommon but clinically important; reported mechanisms include direct infiltration of the anterior chamber angle by neurofibromatous tissue, secondary angle closure from mass effect, developmental dysgenesis of the angle (including congenital ectropion uveae/angle anomalies), and fibrovascularization with subsequent trabecular dysfunction, each of which can lead to raised intraocular pressure and progressive optic nerve damage if not identified early [5]. In children with NF1 who present with periocular plexiform neurofibromas or orbital involvement, clinicians maintain a high index of suspicion and perform thorough anterior-segment evaluation with early glaucoma surveillance [8].

From a molecular perspective, the p.Gln83Ter variant reported in our patient is a truncating (nonsense) mutation predicted to introduce a premature termination codon and cause loss of neurofibromin function via protein truncation and/or nonsense-mediated mRNA decay; truncating and nonsense changes constitute an appreciable fraction of reported *NF1 *pathogenic variants and are well recognized as loss-of-function alleles [9]. While some studies have suggested that certain classes of *NF1* variants (for example, large deletions or some recurrent variants) can be associated with more severe or specific phenotypes, overall genotype-phenotype correlations in NF1 are limited and variable, and thus the presence of a truncating variant alone does not permit precise prognostication [10].

Clinically, this case highlights that children with NF1 benefit from regular, age-appropriate ophthalmic surveillance with careful attention to optic pathway gliomas through visual examinations, behavioral assessments, and targeted imaging when indicated. The presence of periocular or orbital plexiform neurofibromas should alert clinicians to the possibility of secondary glaucoma and anterior-segment involvement, warranting thorough evaluation. Furthermore, early genetic confirmation not only establishes the diagnosis but also enables cascade testing, anticipatory guidance, and coordinated multidisciplinary management involving ophthalmology, clinical genetics, neuro-oncology, and reconstructive services. Together, these measures are essential to preserve vision and optimize long-term outcomes in affected children.

## Conclusions

This case highlights an atypical presentation of NF1, characterized by bilateral optic pathway gliomas confirmed at age 1, and severe, early onset secondary glaucoma in the left eye. Unlike the typical NF1 phenotype, which rarely included glaucoma, this patient exhibited extensive peripheral anterior synechiae in all quadrants with ectropion uveae, as well as a markedly increased axial length, resulting in significant anisometropia. The visual outcome was poor due to severe anisometropic amblyopia, a less commonly reported complication.

Genetic analysis identified a rare truncating *NF1* mutation (c.247C>T, p.Gln83Ter) segregating within the family. Although genotype-phenotype correlations in NF1 are generally limited, truncating variants are often linked with more severe disease, consistent with the extensive ocular findings in this patient. A major limitation of this study was the difficulty in detecting certain *NF1* mutations due to the gene’s extensive heterogeneity and the technical constraints of the diagnostic methods employed.

This case highlights the importance of early genetic confirmation, vigilant ophthalmic surveillance, and coordinated multidisciplinary care to preserve vision and optimize long-term outcomes in children with NF1.
